# Usage of a Digital Health Workplace Intervention Based on Socioeconomic Environment and Race: Retrospective Secondary Cross-Sectional Study

**DOI:** 10.2196/jmir.8819

**Published:** 2018-04-23

**Authors:** Conor Senecal, R Jay Widmer, Kent Bailey, Lilach O Lerman, Amir Lerman

**Affiliations:** ^1^ Mayo Clinic Rochester, MN United States

**Keywords:** race and ethnicity, socioeconomic position, computers, health services research, health disparities

## Abstract

**Background:**

Digital health tools have been associated with improvement of cardiovascular disease (CVD) risk factors and outcomes; however, the differential use of these technologies among various ethnic and economic classes is not well known.

**Objective:**

To identify the effect of socioeconomic environment on usage of a digital health intervention.

**Methods:**

A retrospective secondary cross-sectional analysis of a workplace digital health tool use, in association with a change in intermediate markers of CVD, was undertaken over the course of one year in 26,188 participants in a work health program across 81 organizations in 42 American states between 2011 and 2014. Baseline demographic data for participants included age, sex, race, home zip code, weight, height, blood pressure, glucose, lipids, and hemoglobin A_1c_. Follow-up data was then obtained in 90-day increments for up to one year. Using publicly available data from the American Community Survey, we obtained the median income for each zip code as a marker for socioeconomic status via median household income. Digital health intervention usage was analyzed based on socioeconomic status as well as age, gender, and race.

**Results:**

The cohort was found to represent a wide sample of socioeconomic environments from a median income of US $11,000 to $171,000. As a whole, doubling of income was associated with 7.6% increase in log-in frequency. However, there were marked differences between races. Black participants showed a 40.5% increase and Hispanic participants showed a 57.8% increase in use with a doubling of income, compared to 3% for Caucasian participants.

**Conclusions:**

The current study demonstrated that socioeconomic data confirms no relevant relationship between socioeconomic environment and digital health intervention usage for Caucasian users. However, a strong relationship is present for black and Hispanic users. Thus, socioeconomic environment plays a prominent role only in minority groups that represent a high-risk group for CVD. This finding identifies a need for digital health apps that are effective in these high-risk groups.

## Introduction

Cardiovascular disease (CVD) remains the most prominent cause of morbidity and mortality in the world [1]. A disproportionate amount of disease burden affects people from specific ethnic groups and lower socioeconomic backgrounds [2,3]. These disparities permeate all aspects of cardiovascular care, including prevention, diagnosis, treatment, and outcomes [4,5]. Prevention of CVD through risk factor reduction is an effective and valuable strategy for reducing the majority of disease burden [6,7]. In recent years, wide arrays of digital interventions have been created to aid in this effort [8]. These tools, widely described as digital health, have taken a variety of different forms including mobile apps, texting, desktop-based apps, wearables, digital workplace interventions, and virtual reality. The perceived advantages of digital health tools include increased access, streamlined communication, lower patient costs, and an increase in efficiency and value of health care [9]. Many of these tools have yet to be reliably realized and proven through randomized controlled trials, but the current summation of the evidence points toward benefit in reducing cardiovascular risk [8] and potentially CVD outcomes [10].

For all the perceived benefits of digital health, potential limitations remain. Even with the belief that health care will be improved with these tools, there remain concerns that disadvantaged populations such as those of low socioeconomic status, minorities, and the elderly may obtain fewer benefits. Previous work has shown that people with lower socioeconomic status are less likely to proactively seek online health information, use online health care communication, or participate in user-generated online health activities [11,12]. In one large health system, online patient portal usage was found to be markedly decreased in blacks, Hispanics, patients of low socioeconomic status, and the elderly; differences that largely stem from reduced access in these groups [12]. However, patient education, digital literacy, and other factors are possible contributors as well [13]. This trend is especially concerning given the increased prevalence of cardiovascular risk factors in these populations [3,14,15]. The increased CVD risk coupled with the perceived lack of benefit from digital health tools has been termed the “digital divide.” This divide raises the question of whether these tools provide similar benefit in terms of CVD risk reduction to all users, and if further investigation into groups based on both socioeconomic background and ethnic group may provide more insight.

Previous work from our group examined the usage of a digital health intervention (DHI) as part of a workplace health program (WHP) to determine association with cardiovascular risk factors [16,17]. These previous data provided evidence that DHI usage and benefit, particularly with regard to weight loss over one year, was dependent upon race. In an effort to further explore the digital divide, we utilized publicly available data from the American Community Survey (ACS) cross-referenced with the WHP in a retrospective secondary cross-sectional analysis to determine the relationship between income and DHI use through the lens of race, gender, and age. We hypothesized that DHI use would increase with median income and that the relationship would vary among racial and gender subgroups.

## Methods

### Employee Recruitment and Study Parameters

As described previously [16,17], between 2011 and 2014 CareHere, LLC (Nashville, TN) created and implemented an incentive plan for employees to improve health across 81 employers in 42 of the United States, encompassing 30,974 employees from a variety of ethnic backgrounds and in a variety of occupations in governmental, white collar, and blue-collar settings. CareHere, LLC’s onsite clinic vendor managed the individual programs and tracked results both manually and with the Online CareHere Connect Personal Health Assistant designed and produced by Healarium, Inc (Dallas, TX). All employees enrolled in the employer-sponsored health insurance program were offered the opportunity to complete the biometric screening. The DHI software implementations were branded to each employer but were similar in that they covered basics of CVD prevention that were conceived and designed by CareHere. Delivery methods and interventions did not vary by employer. Employees were given the choice to use the available digital health component of the program upon the initial intake but were not consented at the time of entry, as de-identified data were to be used in the analyses. Race was included as a self-identified attribute at the initial visit. The multivariate analysis was limited to the three predominant racial groups (black, Caucasian, and Hispanic), while other racial groups were excluded due to a small sample size, with the largest identifiable group of others being Asian with approximately 200 individuals. The study and consent process were approved by the Institutional Review Board of Mayo Clinic. No extramural funding was used to support this work. The authors are solely responsible for the design and conduct of this study, all study analyses, and drafting and editing of the manuscript.

Standard laboratory blood tests including fasting lipid panels and serum glucose values were assessed at baseline and every 90 days for one year for those participating, and were paid for through their WHP and/or insurance. The patients’ primary health care providers assessed blood pressure, height, weight, and administered the health behavior questionnaires at baseline and every 90 days in a standard fashion. Employees were asked to follow-up with a health care provider every 90 days for the duration of their WHP (at least one year) to review results and see if all health benchmarks set by the employer were met.

### Digital Health Intervention

**Figure 1:**
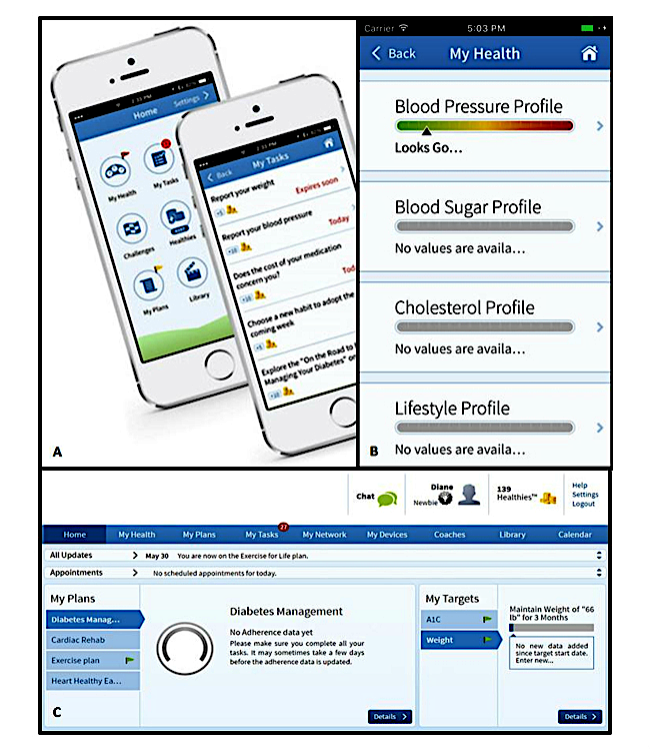
Digital health app mobile and desktop. A) Initial menu and tasks screen of the mobile app; B) My Health visual scales screen within mobile app; C) Desktop version of the digital health intervention. Displayed is the log-in screen that greeted participants following log-in.

The DHI studied here has been described previously [16,17]; the log-in screen is visualized in Figure 1. In brief, it is a platform that is accessible online through a desktop and smartphone-based portal which provides educational materials and concrete health improvement tasks, while the app collects data on the number log-ins to the app via mobile or desktop platforms. Upon enrollment, participants upload baseline data and additional data is compiled as they progress through the program. The platform provides individualized care plans based on medical comorbidities such as obesity, hyperlipidemia, and diabetes, including health status information tasks, targets, and plans that encourage the adoption and maintenance of a healthier lifestyle for improved wellness without physician intervention. Communication occurs directly in the app on a smartphone or desktop as well as through email and short message service (SMS) text messaging. These means of communication can be tailored to user preference in the app’s settings. The number of instances an individual logged into the app throughout the one year studied (log-in frequency) was recorded; this did not include email or SMS messages sent from the app to the user. There were no requirements for log-in frequency. The software directly communicates with an electronic health record that captures International Classification of Diseases codes, medical diagnoses, medical information, demographic information, and lab and vital sign values.

### Socioeconomic Data

The ACS is an ongoing statistical survey conducted by the United States Census Bureau [18] that has been used extensively in clinical research in a similar context [19,20]. The ACS is sent to approximately 3.5 million Americans annually and collects information regarding ancestry, educational attainment, income, language proficiency, migration, disability, employment, and housing characteristics. The ACS is the largest survey administered by the Census Bureau aside from the decennial census. Aggregate data from the ACS is publicly available through American Fact Finder [18]. A query was placed to determine 5-year median income information by zip code from 2008-2013. These zip codes were cross-referenced with the residential zip code for the study participants in order to obtain the residential median income for each participant.

### Statistical Analyses

Statistical analyses were performed by an independent statistician (KB). Baseline characteristics were summarized by frequency percent or by mean and standard deviation and were compared between ethnic groups by analysis of variance (ANOVA) tests or by chi-square tests. Log-in frequency was analyzed in two ways. First, simple and multiple linear regression was used to predict log (1+ “number of log-ins”) as a function of demographic variables: age, sex, ethnicity, and income. A model with only main effects, as well as a second model including all 2-way interactions, was fit. The second method, for verification only, involved a Poisson regression model with “log-ins” as the count variable to be predicted, with a logarithmic link function. The same models with main effects only, and including 2-way interactions, were used.

## Results

Baseline demographics of participants from Caucasian, black, Hispanic, and other groups are shown in Table 1. Participants were predominantly Caucasian (22,278/30,953, 71.97%) and represented 42 separate states. Actual differences between the groups in baseline characteristics were small, but statistically significant differences were seen in all categories except gender and low-density lipoprotein cholesterol measurement. The cohort was found to represent a wide range of socioeconomic backgrounds from a median income of US $11,000 to $171,000 from 2530 separate residential zip codes. The lowest median income of US $11,021 represented an area of Chattanooga, TN and the highest represented Southlake, TX with a median income of US $170,975.

**Figure 2:**
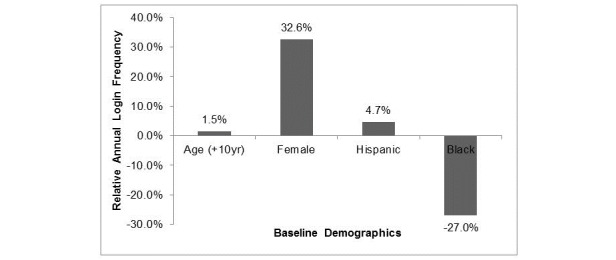
Relative log-in frequency based on demographic variables. The effect of individual demographic variables on log-in frequency to the digital health application. All values are reported as relative changes in log-in frequency, with age compared to 10 years younger, females being compared to males, and with Hispanic and black groups being compared with Caucasians.

In an additive model, all four demographic variables yielded significant independent effects on log-in frequency; this is displayed in Figure 2. Racial groups showed wide variation in average annual log-in frequency. With the Caucasian majority used as a reference, there was an increase in utilization of 4.68% (*P*<.001) in Hispanics, and a decrease of 27.17% (*P*<.001) in blacks. Increasing age was positively associated with DHI log-in frequency, with a 10-year age increase associated with an average of 1.54% (*P*=.003) increase in log-ins. However, this result differed significantly by racial group. Overall, female participants had a 32.59% higher log-in frequency compared to their male counterparts. The interaction of gender and race showed that female gender was consistently associated with increased usage among all racial groups; however, there was considerable variability. Black women showed the strongest gender association with a female to male ratio showing a 18.94% increase and Hispanic women showing a 3.04% increase (both *P*<.001) compared to the female to male ratio in Caucasians.

**Figure 3:**
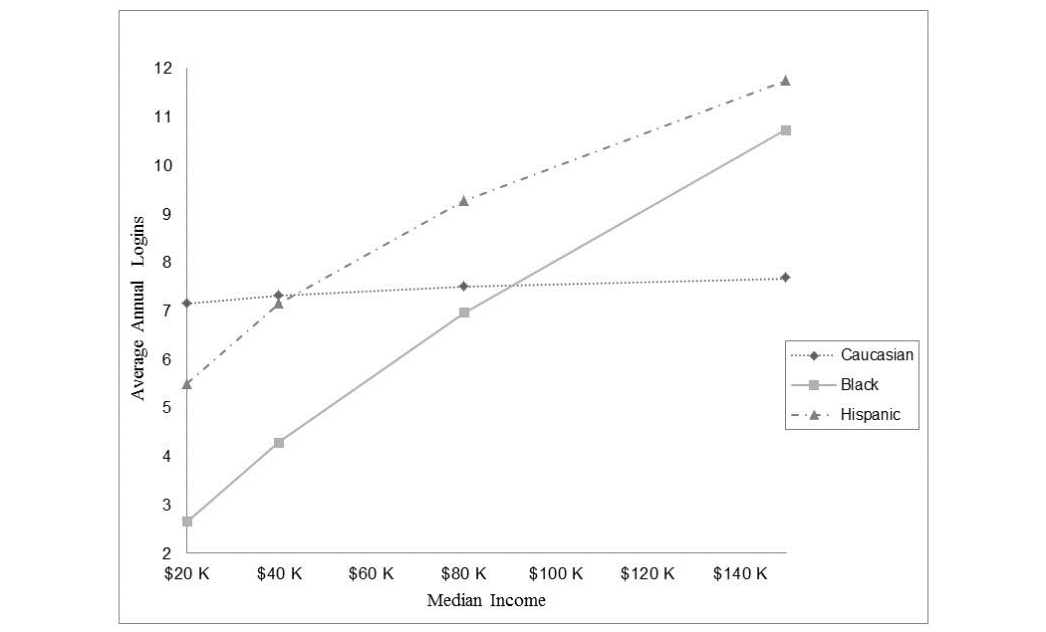
Log-in frequency as determined by combined effect of race and socioeconomic environment. The relationship of monthly log-in frequency by median income of associated zip code separated by racial group. The Y axis displays annual log-ins to the digital health interventionand the X axis displays median annual income of the associated zip code. Differential effects of race on income were statistically significant (*P*<.001).

Figure 3 displays the relationship between log-in frequency and median income of associated zip code. Caucasian participants showed a very modest nonsignificant (3.03%) increase in usage with a doubling of median income. Black participants showed a much larger increase in utilization related to a doubling of median income (40.48%; *P*<.001). Similarly, Hispanic participants also saw a larger increase of monthly log-ins per doubling of median income of 57.8% (*P*<.001). Differences between each group were statistically significant. Median income by associated zip code had separate effects in male and female groups, with females showing a 14.73% (*P*<.001) smaller effect of a doubling of income compared to males.

**Table 1:** Baseline characteristics of workplace health program participants. Cardiovascular risk factors were those collected at initial enrollment visit. Continuous variables are shown with standard deviation. *P* values were derived with ANOVAs to compare means for continuous variables. Chi-square tests were used to compare proportions for categorical variables.

Parameter	Caucasian	Black	Hispanic	Other	*P* value
Participants (n)	22,278	2698	1212	4765	
Age, years (SD)	48.6 (11.3)	48.2 (11.0)	45.0 (11.0)	46.2 (13.4)	<.001
Sex, male (%)	42	44	41	43	.11
Weight, pounds (SD)	197 (50)	214 (51)	193 (57)	190.8 (50)	<.001
Waist circumference, inches (SD)	37.3 (7.0)	39.3 (7.4)	37.1 (6.5)	36.7 (7.1)	<.001
Body mass index, kg/m^2^ (SD)^a^	30.6 (7.2)	33.3 (7.7)	31.6 (6.9)	29.8 (6.9)	<.001
Systolic blood pressure, mmHg (SD)	123 (14)	127 (15)	122 (14)	122 (15)	<.001
Diastolic blood pressure, mmHg (SD)	77 (9)	80 (10)	78 (10)	77.4 (10)	<.001
Triglycerides, mg/dL (SD)	140 (100)	104 (67)	155 (106)	135.8 (84)	<.001
Low-density lipoprotein cholesterol, mg/dL (SD)	111 (32)	112 (33)	111 (32)	111 (33)	.64
High-density lipoprotein cholesterol, mg/dL (SD)	52.1 (15.1)	53.6 (14.1)	49.8 (13.8)	51.8 (15.2)	<.001

^a^Windsored at body mass index: 70

## Discussion

This retrospective secondary cross-sectional analysis demonstrated that socioeconomic status, as derived from the zip code of residential addresses, had a significant effect on digital health usage in this large cohort of working adults. Among Caucasians, the frequency of DHI log-ins showed a small nonsignificant increase as socioeconomic status increased. In comparison, Hispanic participants saw a>50% increase in log-in frequency with a doubling of income and black populations displayed a>40% increase. The current study may have significant implications for future design and implementation of digital health and personalized health care delivery.

Previous studies have identified decreased digital health engagement in people of low socioeconomic status [11,21,22] and in racial minorities [11,23]. Our study reaffirms these findings in individuals of both low socioeconomic status and minority racial backgrounds. However, our study is the first to identify a differential relationship between increasing socioeconomic status and utilization according to race; it is also the first to identify minorities of high socioeconomic status to be more frequent digital health users than their Caucasian counterparts.

These results expand upon our previous DHI findings and demonstrate that baseline racial and socioeconomic characteristics were predictive of DHI log-in frequency [16,17]. Furthermore, female sex was strongly associated with a significant increase in log-in frequency. This correlation had been identified in previous digital health usage studies that addressed online health information seeking and health care communication [24]. Kontos et al examined the Health Information National Trends Survey and found that women were more likely to use health care and user-generated content domains [11]. The mechanism behind these findings may be increased engagement in online health care activities as well as increased use of social media by women as a whole [25,26]. In contrast to previously published work [11,23], but in line with our previous work in this cohort, age in our study was associated with increased DHI across ages from 23 to 88 years. While the increased usage was modest at 1.54% for every decade of age increase, it was notable that the association was positive. This result may be because our study focused on a working age population, which is a group that is more likely to be digitally literate. Individuals beyond working age may not show this increased usage due to decreased access or diminished digital literacy [26,27]. The disparity in log-in frequency by racial groups is especially notable for the highest log-in frequency being among Hispanic participants. Given that increasing age and racial minorities are known associations with worsening cardiovascular risk factors, these new findings may have implications on the use of digital health-based interventions moving forward. For example, future apps may need to be more individually tailored based on underlying user demographics.

This study adds to a growing body of evidence that socioeconomic status affects the utilization of digital health tools. It is notable that the populations in this study were working adults: not included were jobless, disabled, or retired individuals; populations that are likely to have lower incomes and more medical comorbidities. Even with this healthier and more affluent portion of the population, socioeconomic status appears to play a significant role in the utilization of this digital health tool. More work is needed to assess a possible mechanism for this finding, however digital health literacy is a possible consideration. Previous research has documented low digital health literacy in these groups [13,26,27]. The fact that our study took place as part of a WHP also raises the question of whether this relationship had an effect on DHI usage. Although all Health Insurance Portability and Accountability Act requirements were followed, participants could have perceived poorer health as a threat to job security. Future research into user satisfaction and concerns is necessary to further elucidate whether this factor generated an important effect on the results seen in the study.

To our knowledge, no previous study has directly investigated the differing effectiveness of digital health tools based on race and income simultaneously. While more mechanistic studies are needed to elucidate a cause-effect relationship, this study clearly adds to the growing body of evidence describing a digital divide within low-income minorities. The increased usage among minorities of a more affluent socioeconomic background raises several pressing questions about the future of digital health tools. Further research may be able to identify the roots of this gap and find ways to apply these findings to minority groups of lower socioeconomic backgrounds. While true that the design and usability aspects of DHIs should be focused on real-world usability studies with socioeconomic variables as secondary considerations, these data underscore the importance of inclusion in DHI design and implementation.

This study relies on data from the ACS, which provides median income by zip code for individuals in the study, that has been used in a similar fashion previously [19,20]. These values approximate the individuals' socioeconomic environment but are an imperfect correlate for incomes. The use of ACS data assumes an identical median income for all participants with the same residential zip code. A better measure of income may be through participant survey; however, this was not conducted at the time of the study. Follow-up for this study was limited to one year and the durability of these findings over a longer duration are unknown. Future research to investigate causal mechanisms for these observations is necessary to realize equitable gains from DHIs in the workplace and digital health as a whole.

In conclusion, the combination of socioeconomic background and race has a dynamic effect on digital health tool usage in a working adult population, with black and Hispanic populations showing a positive association with increased affluence. Future research may be directed at leveraging high-use affluent minorities to extrapolate strategies to bridge the digital divide in low-income minorities.

## Abbreviations

ACS: American Community Survey
CVD: cardiovascular disease
DHI: digital health intervention
SMS: short message service
WHP: workplace health program
